# Learner feedback and educational outcomes with an internet-based ambulatory curriculum: a qualitative and quantitative analysis

**DOI:** 10.1186/1472-6920-12-55

**Published:** 2012-07-12

**Authors:** Stephen D Sisson, Darius A Rastegar, Mark T Hughes, Amanda K Bertram, Hsin Chieh Yeh

**Affiliations:** 1 Department of Medicine, Johns Hopkins University, 1800 Orleans Street, Baltimore, MD, 21205, USA

**Keywords:** Online education, Curriculum development, Feedback, Learner satisfaction

## Abstract

**Background:**

Online medical education curricula offer new tools to teach and evaluate learners. The effect on educational outcomes of using learner feedback to guide curricular revision for online learning is unknown.

**Methods:**

In this study, qualitative analysis of learner feedback gathered from an online curriculum was used to identify themes of learner feedback, and changes to the online curriculum in response to this feedback were tracked. Learner satisfaction and knowledge gains were then compared from before and after implementation of learner feedback.

**Results:**

37,755 learners from 122 internal medicine residency training programs were studied, including 9437 postgraduate year (PGY)1 residents (24.4 % of learners), 9864 PGY2 residents (25.5 %), 9653 PGY3 residents (25.0 %), and 6605 attending physicians (17.0 %). Qualitative analysis of learner feedback on how to improve the curriculum showed that learners commented most on the overall quality of the educational content, followed by specific comments on the content. When learner feedback was incorporated into curricular revision, learner satisfaction with the instructive value of the curriculum (1 = not instructive; 5 = highly instructive) increased from 3.8 to 4.1 (p < 0.001), and knowledge gains (i.e., post test scores minus pretest scores) increased from 17.0 % to 20.2 % (p < 0.001).

**Conclusions:**

Learners give more feedback on the factual content of a curriculum than on other areas such as interactivity or website design. Incorporating learner feedback into curricular revision was associated with improved educational outcomes. Online curricula should be designed to include a mechanism for learner feedback and that feedback should be used for future curricular revision.

## Background

One of the first steps in curriculum development is a needs assessment of targeted learners [1,2]. Kern et al. state that a curriculum that does not address the needs of its learners risks being inefficient or ineffective [1]. The role of a needs assessment does not vanish once a curriculum has been implemented or is moved online. However, many online curricula have been developed without attention to the principles of curriculum development [3,4]. This is unfortunate, because the Internet adds the capability of efficiently performing recurrent needs assessments of its learners [5]. By incorporating such assessment into design of online curricula, educators can easily determine whether the needs of learners have been met and gauge program effectiveness [5]. This information then serves as the needs assessment for the next round of curricular revision. Many online curricula, however, do not include outcomes assessment and risk becoming out of date [4,6]. Since a curriculum’s goals and objectives will likely evolve over time as learning needs change, these rounds of outcomes assessment, needs assessment, and curriculum revision become important to the long-term success of a curriculum [2].

In addition to enhanced capabilities with outcomes assessment, the Internet offers tools that can be used when educating physicians. As Cook and Dupras state: “The most effective websites creatively integrate content with the power and flexibility of the Web to enhance learning rather than merely replicate traditional methods.” [7] Interactivity is one tool available to Web educators that cannot be replicated in a textbook [5,8,9]. The Internet also allows for the incorporation of multimedia into content, including audio narration, animation, and video clips [7,10,11]. Hyperlinks can be used to augment content, directing learners to source material or additional resources [7]. The impersonality of online training can be minimized by allowing for communication with instructors or other learners [5,7,9,11,12].

With all that is possible with online education, much has been written about what online learners prefer. Atreja et al. showed that the best predictors of satisfaction among the web-based learners they studied were the appeal of the website design and the ability of the course to improve subject understanding [12]. They also found that the ability to communicate with instructors and other learners was associated with greater satisfaction [12]. Others have shown learner satisfaction with web-based education increases when an online curriculum is interactive, with a user-friendly interface that is easy to navigate [5]. Still others have shown that the quality of the online educational content is most important to learners [13]. Many of the features made possible by online learning increase learner satisfaction, but at the expense of an increased time commitment among learners [10].

Internet-based educational programs should be designed and studied to determine those features that learners prefer, ideally incorporating this information into a needs assessment for curricular revision to improve educational outcomes [2,11,14,15]. Heeding that call, we describe here learner feedback on a widely-used online curriculum on ambulatory care and evaluate associated changes in learner satisfaction and educational outcomes.

## Methods

### Data generation

Study data was generated by the Internal Medicine Curriculum on the Johns Hopkins Internet Learning Center. The Johns Hopkins Internet Learning Center is an educational website established in 2002 and is offered to internal medicine residency training programs that subscribe for an annual fee [16]. Residents and faculty at subscribing programs can then access educational content, which is divided into topic-specific training modules. Training modules are structured in a pretest-didactics-post test format. Learners comment on the website and curriculum on general message boards contained on the Internet Learning Center website. Starting in the 2003/04 academic year, learners were required to rate the instructive value of the module on a Likert scale (1 = not instructive; 5 = highly instructive). Starting in the 2005/06 academic year, learners were also required to give feedback on each training module by answering in free text the following: “Please tell us how to improve this module”. Learners could enter more than one comment or even nonsensical comments. For this study, feedback data (i.e., module instructive value ratings and free text comments) were analyzed from the 2003/04 academic year through the 2007/08 academic year on 19 training modules that were part of the curriculum each of those academic years. For feedback comments, 1000 comments were randomly selected for each of academic years 2005/06, 2006/07, and 2007/08. Likert ratings on instructive value from all learners who completed any one of the 19 training modules were included for study. The study was approved by the Johns Hopkins Institutional Review Board.

### Data analysis

Three team members (SS, DR, and MH) read all 3000 feedback comments to identify main themes. Themes were used to develop a coding sheet to qualify comments on a goodness/badness, quality/quantity scale as well as on the aspect of the curriculum (e.g., questions, content or website function) addressed by the comments. The goodness/badness/quality/quantity scale categorized comments as either “bad”, “good”, “both good and bad”, “neither good nor bad”, “too short/too few”, “too long/too many”, “quantity just right”, or “additional requested features”. The curricular aspect/website function scale comments were categorized as no meaningful response, overall quality of the module, factual content of module, content organization, module images, module length, question quality, question quantity, or website function. The same three team members then re-read each feedback comment and coded them on each of the two categorization aspects. Unanimity among all three coders was required; when unanimity was not present, feedback comments were discussed until consensus was reached.

Average change in scores was calculated by subtracting aggregate pretest scores from aggregate post test scores for each module. Reliability testing was done on all pretests and post tests by performing item discrimination on each item and Cronbach’s alpha on each test. Content of pretests and post tests underwent face validity testing, and test results were evaluated by training year to perform construct validity. Reliability and validity testing on this curriculum has been reported elsewhere [17]. These scores were then weighted by the number of learners for each module in a given academic year. Then the average change in scores was determined among all modules in a given academic year. We used frequency analyses to describe the respondent population and comment themes, Student’s t-test to compare average module ratings, and one-way ANOVA to compare average change in module score. Tests of significance were two-tailed, with an alpha level of 0.05. We performed analyses using SPSS, version 18.0 (Chicago, IL).

## Results

### Description of learners

All learners were affiliated with internal medicine residency training programs that subscribed to the Johns Hopkins Internal Medicine Curriculum through the Johns Hopkins Internet Learning Center. Subscribing programs included academic medical centers, community hospitals, and military hospitals. In academic year 2003/04, there were 3670 learners at 50 subscribing programs, which grew to 10,235 learners at 122 subscribing programs by the 2008/09 academic year. During the academic years 2003/04 through 2008/09 a cumulative 37,755 learners registered with the website. Most learners were internal medicine resident or attending physicians (Table 1), but included a small number of medical students, administrative staff, or undefined users (grouped as “Other”). Subscribing programs uniformly required that resident physicians complete any number of the 19 modules studied, while completion by attending physicians was voluntary. Learners completed a total of 128,850 modules over the six years of study.

**Table 1:** Learner demographics

**Year of Training**	All learners* N (%)	**Qualitative feedback**^**+**^**analysis N (%)**
**PGY1**	9437 (24.4)	1019 (34.0)
**PGY2**	9864 (25.5)	926 (30.9)
**PGY3**	9653 (25.0)	852 (28.4)
**Attending**	6605 (17)	64 (2.1)
**Other**	3196 (8.2)	139 (4.6)
**Total**	37,755	3000

*Academic years 2003/04-2008/09; ^+^Academic years 2005/06 -2007/08.

Website capability to gather qualitative feedback from learners was added in the 2005/06 academic year, and was a mandatory component of module completion. For the purposes of this study, a subset of 1000 comments was gathered from each of the first three academic years that qualitative feedback was available. The training year or attending status of learners used in the random sample of 3000 feedback comments is shown in Table 1.

### Qualitative analysis

Thematic analysis of learner feedback identified five themes of learner comments: the overall quality of the module (mentioned in 32.7 % of comments), the content of the module (16.0 % of comments), the questions contained in the module (4.6 % of comments), the length of the module (0.7 % of comments), and website function (2.4 % of comments) (Table 2). Further breakdown of specific comments is shown in Table 2. Nearly all comments on the overall quality of the module were positive. Of the specific comments on content, almost half were concerns about the factual content of the curriculum, followed by praise of the factual content. The remainder of specific comments on content was on organization and images, with the majority of comments requesting more summary tables, charts and images.

**Table 2:** Qualitative feedback

**Subject**	**N (%)**	**Examples**
**Overall quality of module**	1026 (32.7)	
Good	1008 (32.2)	· Awesome module · A very good learning module · Good
Bad	18 (0.6)	· Proofread it; there are numerous grammatical errors throughout the module · Found this module to be minimally helpful · Some mistakes
**Module content**	503 (16.0)	
Factual good	154 (4.9)	· Informative · Good review on diabetes treatment · Good review of cancer screening
Factual bad	219 (7.0)	· Would like to hear more about recent studies · Include trade names with generics to make it easier to comprehend · The use of bisphosphonates was not entirely clear
Length/ organization good	55 (1.8)	· Liked the diagrams · This module was done very well in a stepwise manner · Liked the format of the clinical cases as well as the pretest/post test organization
Length/organization bad	32 (1.0)	· More printable tables · More charts · Summarize “red flags” of back pain somewhere in the module
Images/too few	43 (1.4)	· This module could be improved by having pictures · More pictures · Pictures could have been included (e.g., tonsillar exudates, local GABHS complications, scarlet fever rash)
**Module questions**	144 (4.6)	
Quality good	18 (0.2)	· Very good set of questions · Good questions · Very complete in its questions
Quality bad	47 (1.5)	· The pretest questions were poorly worded · Very poor questions; answers do not correspond with information given in the module · You can add more clinical settings to the stem of the questions
Quantity bad	79 (2.5)	· Increase practice questions · Ask more questions · Less questions
**Module length**	22 (0.7)	
Too long	22 (0.7)	· Make explanations more concise · Too long · Was just too lengthy
**Website function**	75 (2.4)	
Good	3 (0.1)	· Good organized feedback · Easy to use · I like that we can download summaries in PDF
Bad	13 (0.4)	· The case answer was different than the correct answer · I wasn’t receiving credit for correct questions during the cases · The “pop up” had the wrong answer
Additional features requested	59 (1.9)	· Try to time the modules · I think you should give an idea of how many questions are in a section · Brief reading material before the start of the module
**Total**	3135 (100)	

Learner comments on module questions most commonly pertained to the number of questions (usually asking for more of them) followed by complaints about the quality of the questions. For those learners commenting on website function, the majority requested additional website features, including a timer function and a navigation map for each module. The percentage of comments on topics other than the overall quality or content of the modules was low.

### Response to feedback

**Figure 1:**
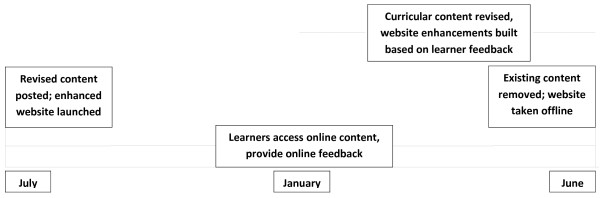
**Annual repeating cycle of feedback-based curricular revision and website enhancement.** A timeline of annual curricular cycle is shown. Learners access curricular content throughout the academic year and provide feedback. Curricular content is revised and website enhancements are designed annually based on learner feedback. Old content is removed at the end of the academic year and revised content and website upgrades are added as the new academic year begins.

In response to specific feedback from learners, revisions were made annually to curricular content and website design/function was enhanced (Figure 1 and Table 3). Curricular content was revised to make it more visual, with greater numbers of tables, figures, algorithms, and images. A small number of demonstration videos were added. The number of pretest and post test questions was increased and explanatory text for answers on the pretest and posttest were added. When appropriate, didactic sections were summarized with recap statements at the end of the section. Website design and function was changed to add a navigation map to each module. The didactics section was changed so that learners could do the didactic sections in the order of their choosing (i.e., “learner control”), although they had to complete the pretest before the didactics, and could not access the post test until after completing all the didactics (i.e., “program control”). Finally, factual content of modules was updated annually irrespective of feedback comments when necessitated by advances in the medical literature.

**Table 3:** Summary of curricular and website changes

**Sphere**	**Changes (year added)**
**Curricular content**	· Increased number of tables (all years) · Increased number of figures/algorithms (all years) · Increased number of images (all years) · Increased number of questions on pre/post tests (all years) · Added explanatory text on correct/incorrect answers on pretest/post test (2007) · Added recap statements at end of didactic sections (all years)
**Website design/function**	· Added navigation map to each module (2007) · Changed didactic section from program control to learner control (2007) Added video content capability (2005) · Added 1-page printable summaries for completed modules (2006)

### Changes in education outcomes

**Figure 2:**
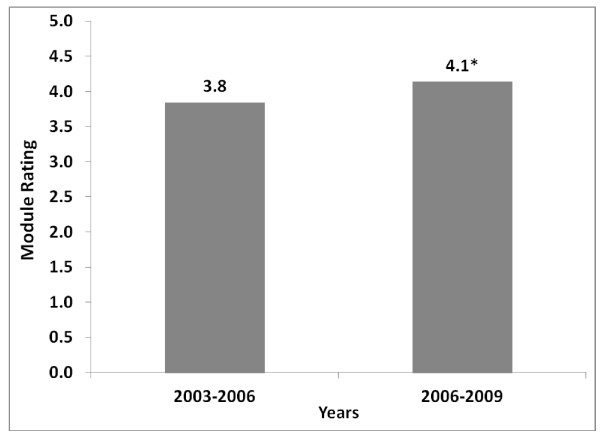
**Average instructive value of modules.** Average learner ratings on instructive value of didactic modules (1 = not instructive; 5 = highly instructive) are compared between the 2003–2006 academic years (before qualitative feedback was incorporated into annual curricular revision cycle) and the 2006–2009 academic years (after qualitative feedback was incorporated into annual curricular revision cycle). *Difference is statistically significant (p < 0.001).

The average module ratings on the instructive value of the 19 modules studied were tracked for the three years before and three years after the incorporation of qualitative feedback in curricular revision (Figure 2). A total of 56,254 modules were completed in the “before” group, with an average instructive value of 3.8. In the “after” group, a total of 72,596 modules were completed, with an average instructive value of 4.1. The difference between these two scores was statistically significant (p < 0.001).

**Figure 3:**
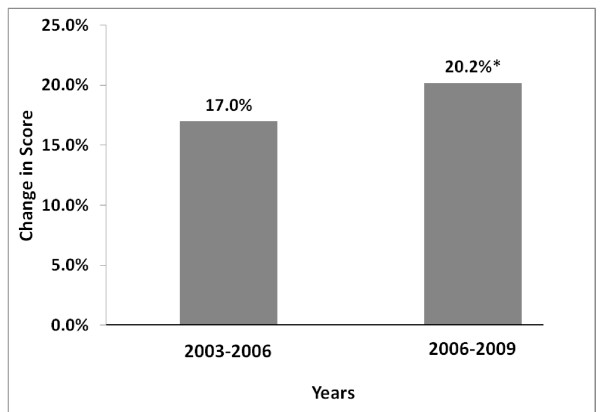
**Average changes in score per module by academic year.** Average change in score (post test minus pretest) on didactic modules are compared between the 2003–2006 academic years (before qualitative feedback was incorporated into annual curricular revision cycle) and the 2006–2009 academic years (after qualitative feedback was incorporated into annual curricular revision cycle). *Difference is statistically significant (p < 0.001).

The average change in scores (i.e., post test minus pretest) was calculated on the 56,254 modules studied in the three years before incorporation of qualitative feedback in curricular revision and is compared with the average change in scores on the 72,596 modules completed after feedback was incorporated in curricular revision (Figure 3). In the “before” group, the average improvement in scores was 17.0 %. In the “after” group, the average improvement in scores was 20.2 %. In comparing these two groups of changes in scores, these differences were statistically significant (p < 0.001).

## Discussion

Using qualitative feedback from learners as a needs assessment to guide curricular revision and website design is associated with greater learner satisfaction and larger gains in knowledge. Qualitative learner feedback on desired improvements centers mostly on the general quality of the module rather than any specific component of the content or website function. When making specific comments, the greatest number of comments is on the educational content of the curriculum, particularly its factual elements. A small proportion of learners request reorganization of content, typically to include more tables, charts, figures, and images. Those who comment on practice questions typically want more of them. A small number of learners comment on website function, usually to request additional features.

While others have commented that taking bad educational content and putting it online doesn’t improve learner satisfaction, we found that responding to learner feedback on content that is generally rated as good and reorganizing that content (i.e., adding tables, figures, and images) and changing website design/function in response to specific feedback improved learner satisfaction [7]. What we found demonstrates the value of recurrent needs assessment in curricular revision. Needs assessment should be done when a curriculum is first developed, but since learners might not fully know their needs until exposed to subject matter, and learner needs evolve, recurrent needs assessment should be built into online curricular design [1,2,4,18]. Over time, our curricular content became more visual (through the addition of tables, figures, and images) and more interactive (through the addition of more questions and greater opportunity for feedback). With these changes, satisfaction among our learners improved. Getting learners to repeatedly access an online curriculum requires that they be satisfied with it [8,18]. Our website design, including a strong evaluation component, and our process of using this information as a recurrent needs assessment, is one way to increase the chances of success of an online curriculum.

While placing a curriculum online offers additional tools to enhance content (i.e., interactivity, hyperlinks, audio and video) our results confirm what others have shown: the quality of educational content is what matters most to learners [7,12,13]. The majority of learner comments on the curriculum were related to the overall quality of the modules and their educational content, suggesting that the educational quality of the content was most important to learners. Learner satisfaction is also strongly associated with knowledge gains. In a meta-analysis of studies of predictors of learner satisfaction done before the Internet was widely used, learner satisfaction with a curriculum was determined most by how much learners felt they got out of the educational content (i.e., “teaching effectiveness”) [19]. In our study, we used the average change in scores on a module (i.e., post test score minus pretest score) as a measure of teaching effectiveness. We found that by responding to learner feedback, the teaching effectiveness of our modules improved. Since teaching effectiveness is such a driver of learner satisfaction, it is not surprising that our results showed similar findings in teaching effectiveness and learner satisfaction. Although we are unable to determine which changes (i.e., website functionality, content organization etc.) contributed to these improvements, it may not matter. The more important finding is that the process of incorporating learner feedback into curricular revision is associated with improved outcomes in learner satisfaction and knowledge outcomes.

It is our opinion that online curricula should be designed to include powerful evaluation tools (including learner satisfaction as described here) to assess the reliability and validity of education outcomes. We have continually leveraged the capabilities of being online to increase the evaluation component of our curriculum. Assessment tools (i.e., pretests and post tests) were expanded, and item discrimination and Cronbach’s alpha calculations were added to these instruments. Group performance measures, subgroup (i.e., training year; training program) performance measures, and individual performance measures (including standard scores) were added or expanded. This evaluation component allowed us not only to perform a continuous needs assessment of our learners, but also to serve the needs of the residency program directors that chose to implement our curriculum. The Accreditation Council of Graduate Medical Education (ACGME) requires that program directors demonstrate the educational outcomes of their curricula, which is provided at a group and individual level by our curriculum [20]. Use of our curriculum has grown from 24 internal medicine residency programs in 2002 to over 160 programs in 2011. As others have pointed out, tracking evaluation outcomes and learner feedback consumes resources, as does the resultant curricular revision and implementation of website improvements, but these costs are minimized by sharing resources among users [4,5]. Developing a strong evaluation component to online curricula allows educators to advance the science of education and answer the call to increase evaluative research on education outcomes and those features that improve them [15,21,22].

Our study has several limitations. Individual learners changed from year to year, and so changes in learner satisfaction and knowledge outcomes may have been due to learner characteristics (e.g., post-graduate year, program type) rather than the curriculum. Since several changes to curricular content and website design were made in any given year, we were unable to measure the impact of any single change on learner satisfaction or knowledge outcomes. Over the years of study, online education resources expanded greatly, and learners may in general have become more satisfied with online didactics independent of our changes. Also over the years of study, clinician educators who wrote the educational content may have improved their writing skills, contributing to improvement in the educational content of the curriculum.

## Conclusions

We found that when asked to give feedback on how to improve an online curriculum, learners commented most on its educational content rather than its interactivity or website design. We also found that a process of recurrent needs assessment followed by curricular revision is associated with improved learner satisfaction and larger gains in knowledge. Online educational curricula should be designed to include evaluative tools that measure educational outcomes for study so that educational outcomes can be improved.

## Abbreviations

ACGME, Accreditation Council of Graduate Medical Education.

## Competing interests

Drs. Sisson, Rastegar and Hughes receive an annual stipend for editorial duties with the curriculum described herein.

## Authors’ contributions

SS, DR, MH, and AB were responsible for the original conception and design of this study. AB was responsible for acquisition of data. AB and HY performed the statistical analysis of the data. SS, DR, MH, AB and HY were responsible for interpretation of the data, drafting of the manuscript, and revision of the manuscript. All authors read and approved the final version of the manuscript.

## Pre-publication history

The pre-publication history for this paper can be accessed here:

http://www.biomedcentral.com/1472-6920/12/55/prepub
